# Supplementary material for: Patient-derived frontotemporal lobar degeneration brain extracts induce formation and spreading of TDP-43 pathology in vivo
Source: Nat Commun. 2018 Oct 11;9:4220. doi: 10.1038/s41467-018-06548-9 (PMC6181940; doi:10.1038/s41467-018-06548-9)
Supplement: Supplementary file 1 — Supplementary Information [file 41467_2018_6548_MOESM1_ESM.pdf]

Supplementary Information

Patient-derived Frontotemporal Lobar Degeneration Brain Extracts Induce Formation and Spreading of TDP-43 Pathology *in vivo*

Porta et al.

Supplementary Figure 1

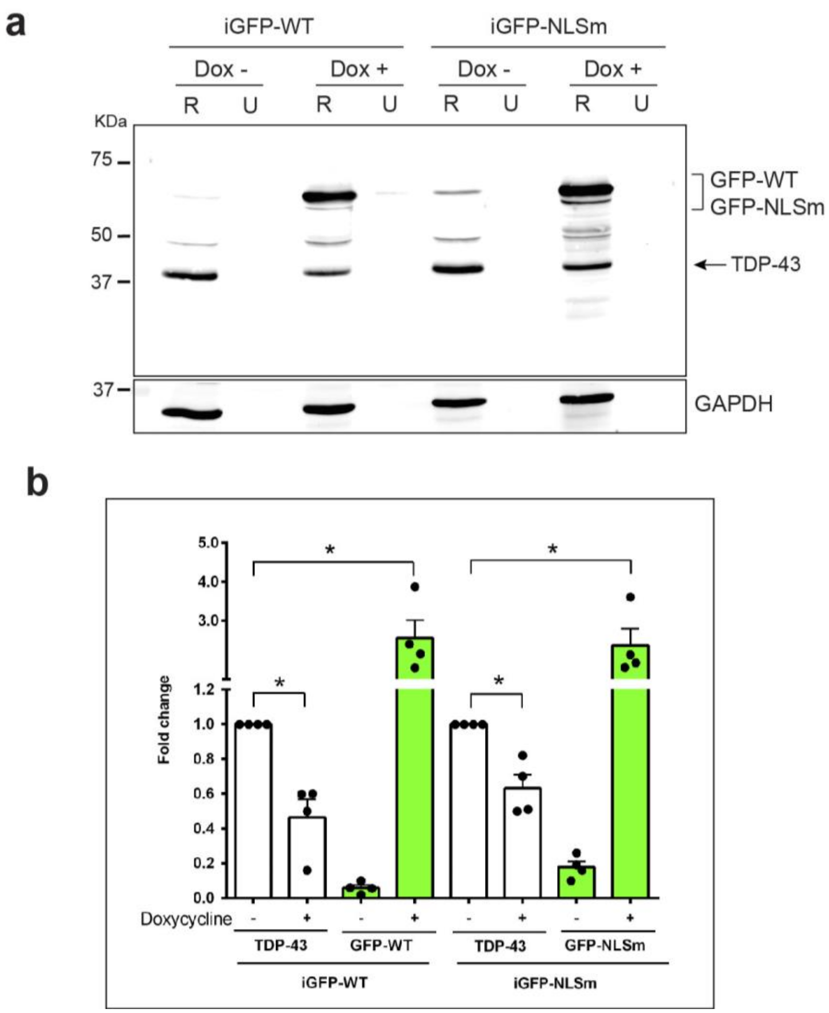

Supplementary Figure 1. Expression of GFP-WT, GFP-NLSm and endogenous TDP-43 proteins in inducible cell lines.

a) Representative immunoblot of protein extracts from iGFP-WT and iGFP-NLSm stable cells sequentially extracted with RIPA (R) buffer followed by UREA (U) buffer from cells grown for 72 hrs in the absence (Dox-) or presence of Dox (Dox+). A pan-TDP-43 antibody was used to detect the endogenous TDP-43 (lanes Dox+ and Dox-, arrow) and both GFP-WT and GFP-NLSm fusion proteins (lanes Dox+). Bottom panel shows immunoblotting for GAPDH protein used as a loading control. **b** Plot shows quantification of the protein levels of endogenous TDP-43 (white bars), GFP-WT and GFP-NLSm proteins (green bars) in the absence (Dox-) or presence (Dox+) of Dox in the RIPA fractions, in both iGFP-WT and iGFP-NLSm stable cell lines. Bar-plots show mean and whiskers s.e.m., with individual points representing independent experiments overlaid as black circles (n=4). iGFP-WT and iGFP-NLSm stable cell lines were analyzed separately and a paired two-tailed Student´s *t*-test was used to compare the levels of GFP-WT, GFP-NLSm and endogenous TDP-43 in presence of Dox (Dox+) versus the endogenous levels of TDP-43 in absence of Dox (Dox-). *P< 0.05.

Supplementary Figure 2

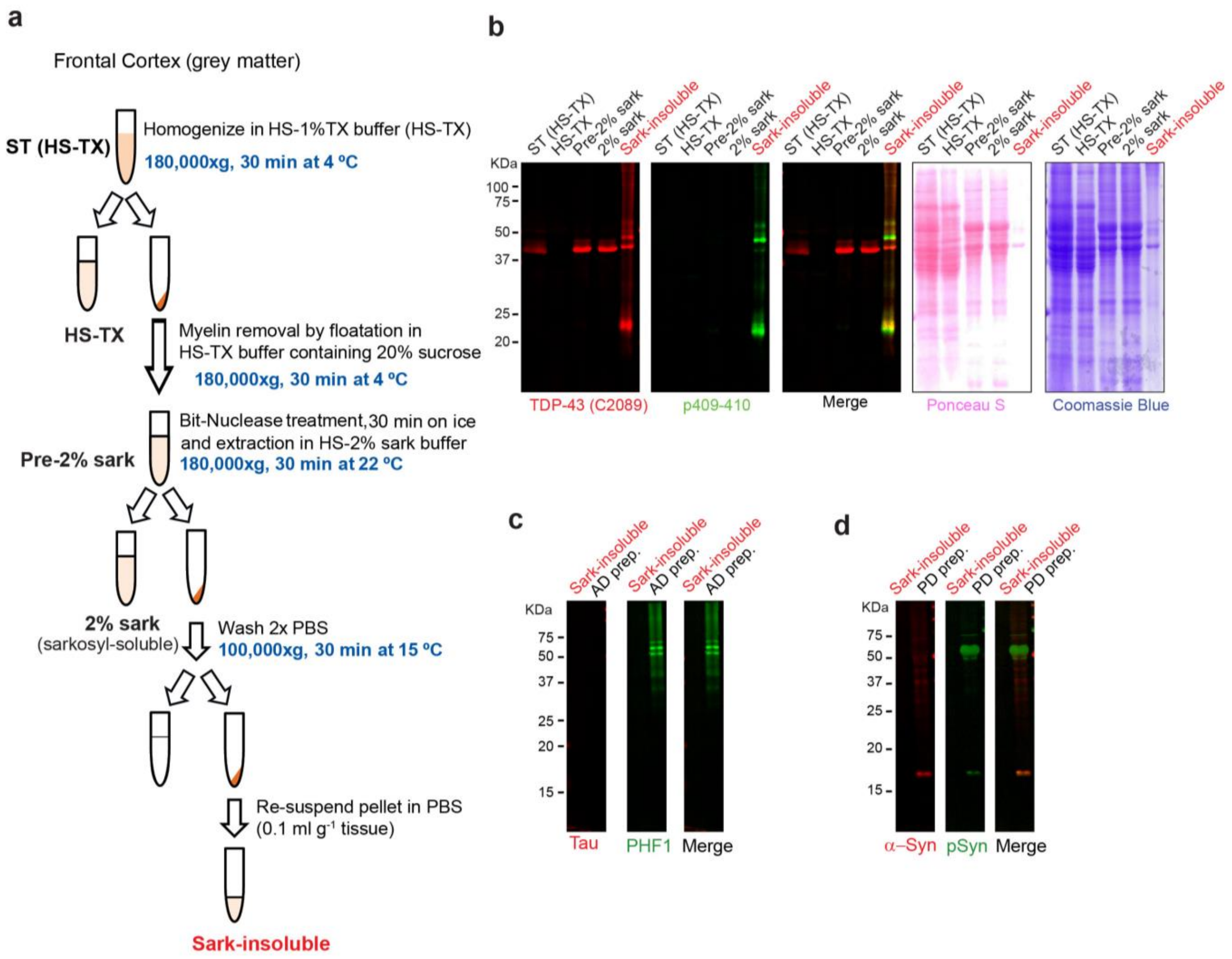

Supplementary Figure 2. Extraction protocol and biochemical characterization of FTLD-TDP and control brain extracts.

a) Schematic diagram of the extraction protocol used to obtain the sarkosyl-insoluble fraction containing TDP-43 protein. Each fraction from a FTLD-TDP brain-extraction was analyzed by immunoblot in **b**: ST (HS-TX) (starting homogenate), HS-TX, Pre-2% sark, 2% sark and the final sarkosyl-insoluble fraction (Sark-insoluble) containing TDP-43 protein was used in this study as pathogenic seeds. A C-terminal TDP-43 antibody (C2089, red) was used to detect total TDP-43 protein and an antibody specific for TDP-43 phosphorylated at Ser409/Ser410 (p409-410, green) was used for detection of the insoluble/pathological TDP-43. Ponceau S and Coomassie Blue staining were used to reveal other protein contaminants. The same sarkosyl-insoluble fraction analyzed in **b** was immunoblotted in **c** using antibodies specific for total tau (red) and hyper-phosphorylated pathological tau (PHF-1, green) and in **d** specific for total α-syn (α-Syn, red) or phosphorylated α-syn (pSyn, green). Enriched tau preparations from an AD brain (AD prep., in **c**) or α-syn enriched from a PD brain (PD prep., in **d**) were used as positive controls.

Supplementary Figure 3

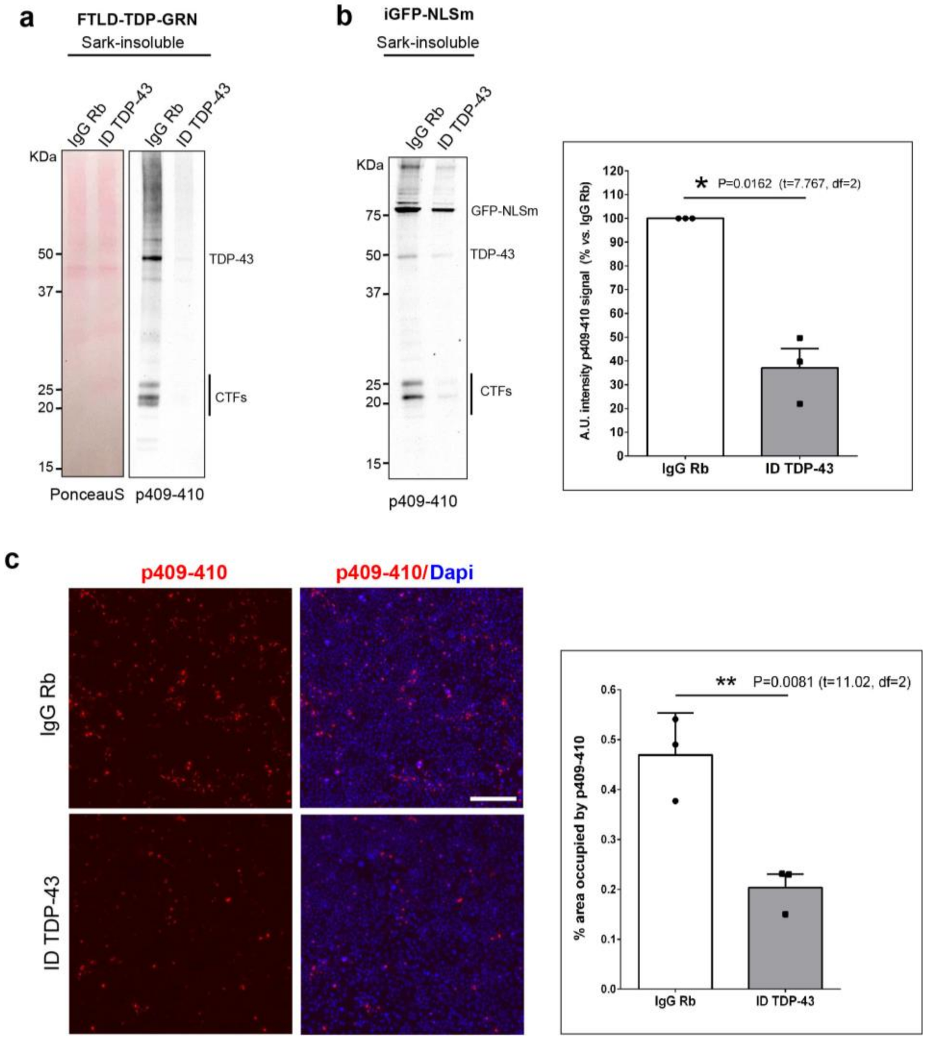

Supplementary Figure 3. TDP-43 immunodepletion from FTLD-TDP extracts reduce seeding activity *in vitro.*

a) Representative immunoblots of unbound proteins in the sarkosyl-insoluble fraction from FTLD-TDP-GRN brain extracts after immunodepletion with a rabbit polyclonal antibody against TDP43 (ID TDP43) or rabbit IgG as a control (IgG Rb). Main protein contaminants detected by Ponceau S red were still present in IgG Rb and ID TDP-43 lanes while immunoblot using the phospho-specific p409-410 antibody shows that full length TDP-43 and the CTFs were largely immunodepleted in ID TDP43 lane. **b)** Representative immunoblots of sarkosyl-insoluble extracts obtained from iGFP-NLSm cells transduced with either IgG Rb (white bar) or ID TDP43 (grey bar) immunodepleted supernantants (described in **a**) at 3 dpt. Plot shows the densitometric analysis of p409-410-immunopositive signal (A.U.) represented as a percentage with respect IgG Rb conditions. Bar-plots show mean and whiskers s.e.m., with individual points representing independent experiments overlaid as black circles (n=3). A paired two-tailed Student's *t*-test was performed. *P<0.05. **c**) Representative IF images show p409-410-positive aggregates in iGFP-NLSm transduced with either IgG Rb or ID TDP43 immunodepleted supernantants (described in **a**). Cells were counterstained with DAPI to label the nuclei. Scale bar = 200 µm. Note dramatic reduction in TDP-43 aggregates in the ID TDP-43 panels. Plots show the quantification of the % of area occupied by p409-410-positive immunostaining in iGFP-NLSm cells transduced with either IgG Rb (white bar) or ID TDP43 (grey bar) immunodepleted supernantants (described in **a**) at 3 dpt. Bar-plots show mean and whiskers s.e.m., with individual points representing independent experiments overlaid as black circles (n=3). A paired two-tailed Student's *t*-test was performed. **P<0.01.

Supplementary Figure 4

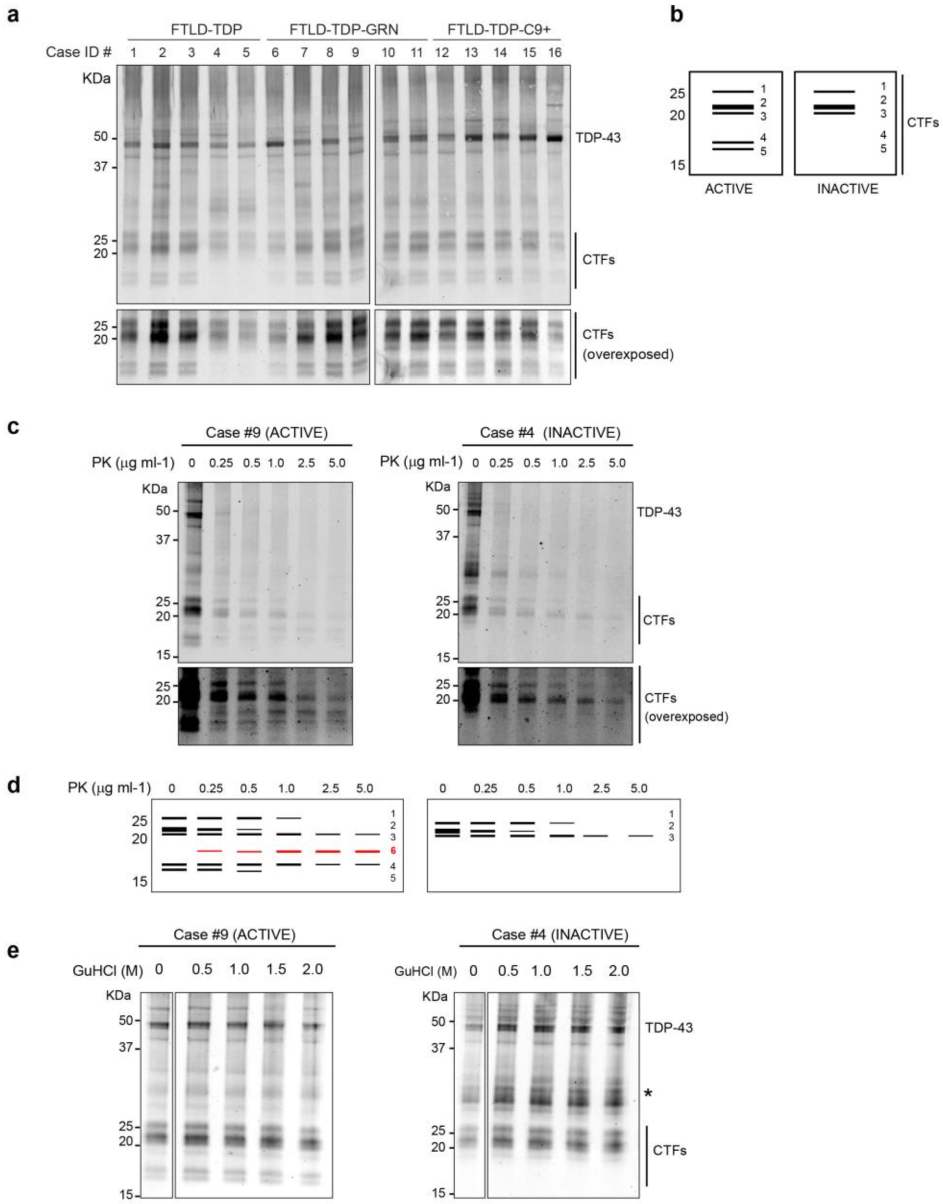

Supplementary Figure 4. Biochemical characterization of sarkosyl-insoluble TDP-43 from FTLD-TDP brains.

a) Immunoblot analysis of the sarkosyl-insoluble fraction (1 ng total TDP-43) from FTLD-TDP cases in our cohort using the C-terminus TDP-43 antibody (C1039). Cases are identified by numbers corresponding to those in Supplementary Table 1 (Case ID #). Molecular weight markers in KDa are shown on the *left* and position of full length TDP-43 protein (~43KDa) and C-terminal fragments (CTFs, ~25-18KDa) on the *right*. Bottom panels show a higher intensity images for the CTFs fragments (overexposed). **b**) Schematic diagram of the two distinct band pattern of the CTFs detected in cases with high seeding activity (i.e., #1-3 and #6-16 in **a**, active) or less active (i.e, #4 and #5 in **a**, inactive). **c**) Representative immunoblots blots using C1039 antibody show the digestion pattern of TDP-43 on the Proteinase-K (PK) assay using active (i.e., case #9 in **a**) or inactive (i.e, case #4 in **a**) brain extracts. The concentration of PK (µg ml^{-1}) used is labeled in each lane. Bottom panels show a higher intensity images for the CTFs fragments (overexposed). **d**) Schematic diagram of the two distinct band pattern of CTFs after PK digestion (in **c**). **e**) Representative immunoblots using C1039 antibody show the banding pattern of TDP-43 protein on the GuHCl assay using active (i.e., #9 in **a**) or inactive (i.e, #4 in **a**) brain extracts. The concentration of GuHCl (M) used is labeled in each lane. Intermediate TDP-43 immunoreactive bands between ~ 25-37 KDa are labeled with an asterisk.

Supplementary Figure 5

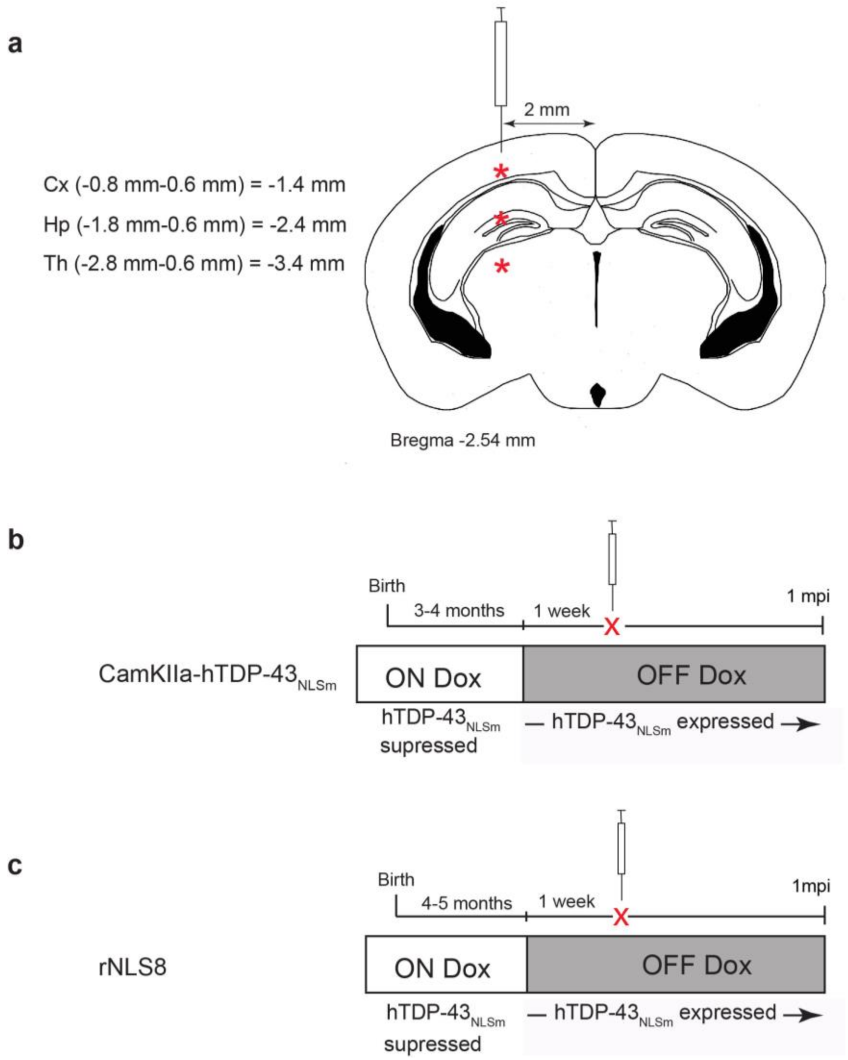

Supplementary Figure 5. Experimental design of stereotaxic injection of CamKIIa-hTDP-43$_{NLSm}$ and rNLS8.

Schematic illustrations of the injection sites (**a**) as well as experimental paradigm for CamKIIa-hTDP-43$_{NLSm}$ (**b**) and rNLS8 (**c**) Tg mice used in these studies.

Supplementary Figure 6

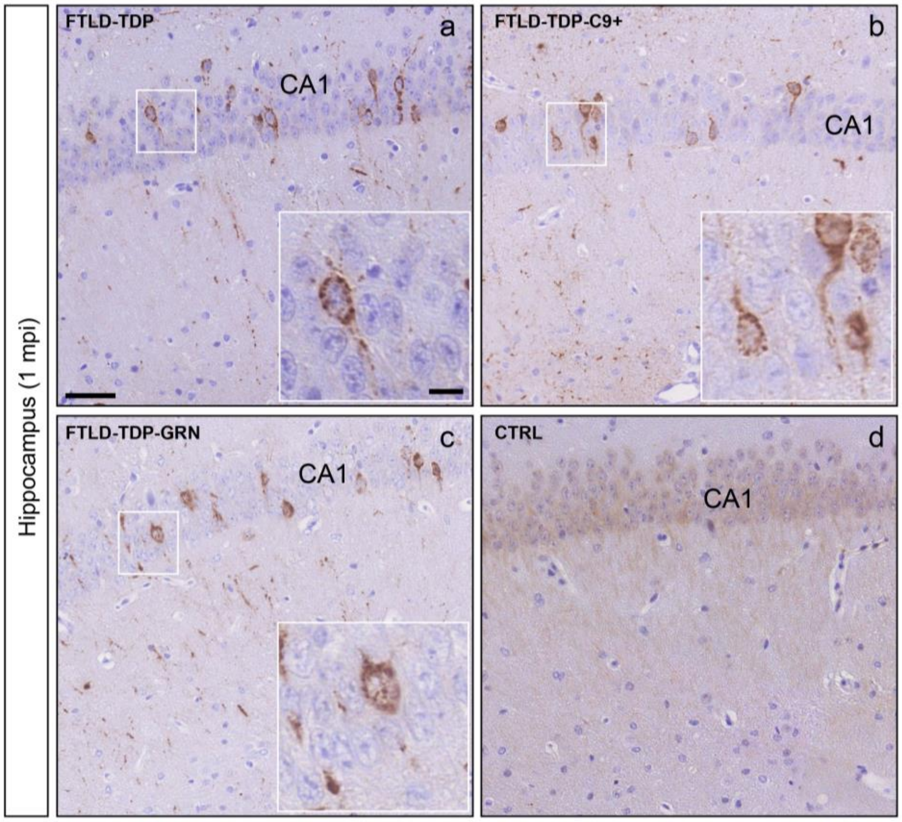

Supplementary Figure 6. TDP-43 seeding with FTLD-TDP extracts and not control extracts. Representative photomicrographs of p409-410 IHC staining in the ipsilateral hippocampus (**a-d**) of CamKIIa-hTDP-43_{NLSm} mice following brain injections with extracts from sporadic FTLD-TDP (n=3) in **a**, FTLD-TDP-C9+ (n=3) in **b** and FTLD-TDP-GRN (n=2) in **c** or age matched non-pathological (CTRL) individuals (n=3) in **d** at 1 mpi. Notice the presence of p409-410 staining in CA1 pyramidal cells in CamKIIa-hTDP-43_{NLSm} mice following brain injections with sporadic FTLD-TDP, FTLD-TDP-C9+ and FTLD-TDP-GRN extracts, but not in mice injected either with CTRL extracts (**d**). Insets are higher magnifications of the white boxes in **a**-**c**. Scale bar = 50 µm (**a**-**d**) and 10 µm (insets).

Supplementary Figure 7

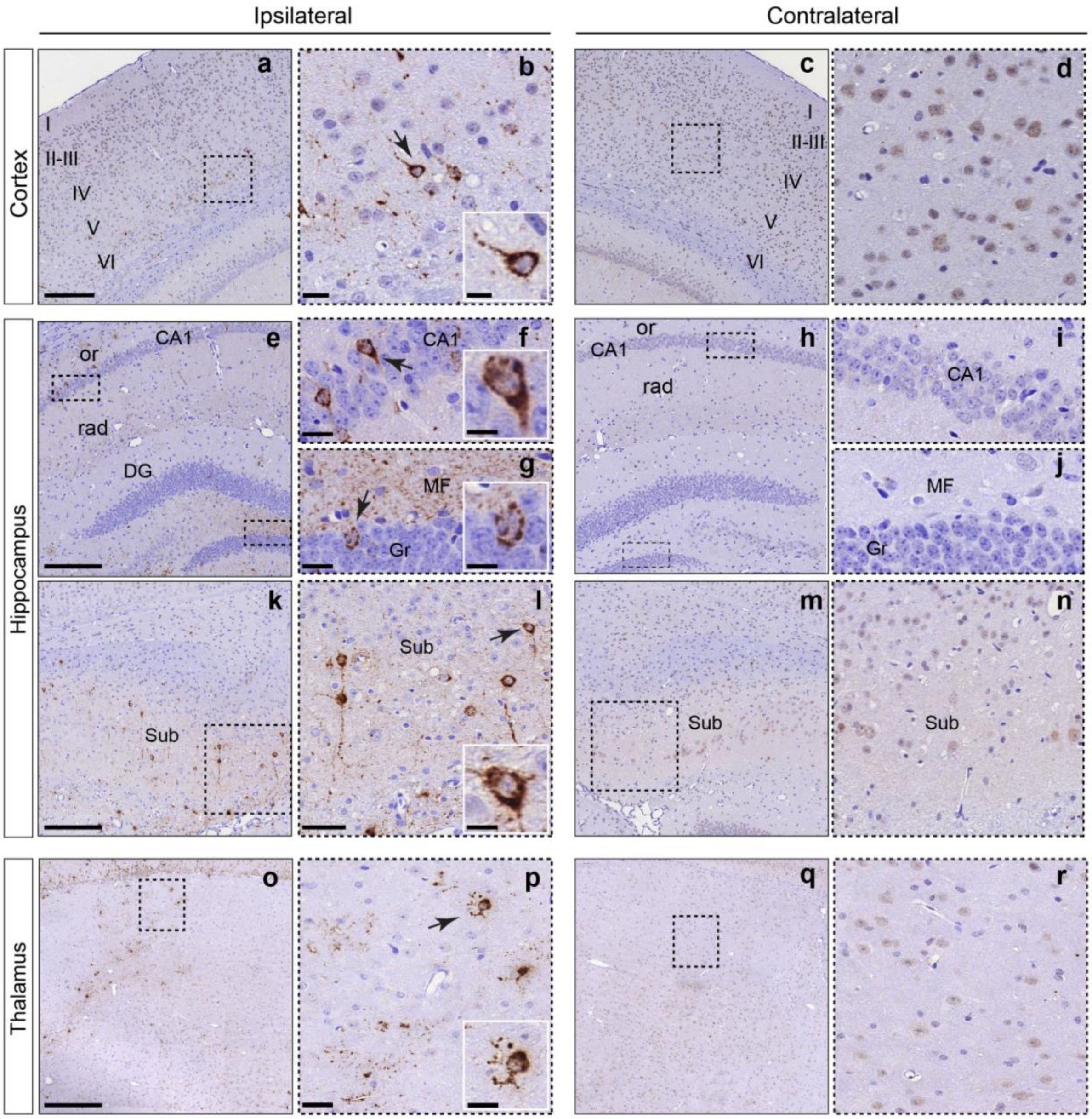

Supplementary Figure 7. Formation of TDP-43 pathology in rNLS8 injected with FTLD-TDP-GRN extracts. Representative photomicrographs of p409-410 IHC staining in cortex (layers I-VI**, a**-**d**), hippocampus (**e**-**n**) and thalamus (**o**-**r**) of injected rNL8 mice at 1 mpi (n=3) in ipsilateral and contralateral sides of the brain. The p409-410-positive staining in deeper layers of the cortex is shown in **b** and **d** as higher magnifications of the black-dashed boxes in panels **a** and **c**. Panels **e**-**n** show p409-410 immunostaining in hippocampus; CA1 layer, stratum radiatum (rad), stratum oriens (or), dentate gyrus (DG) and subiculum (Sub). Higher magnifications of the black-dashed boxes in **e**, **h**, **k** and **m** show CA1 pyramidal cells (**f** and **i**), granule cells (Gr) and mossy fibers (MF) (**g** and **j**) and subiculum (Sub) (**l** and **n**). Arrows point to p409-410-positive cells magnified in the insets (**b, f**-**g**, **l** and **p**). Scale bar = 200 µm (**a**, **c**, **e**, **h**, **k**, **m**, **o** and **q**), 20 µm (**b**, **d**, **f**-**g**, **i**-**j**, **p** and **r**), 50 µm (**l** and **n**) and 10 µm (insets).

Supplementary Figure 8

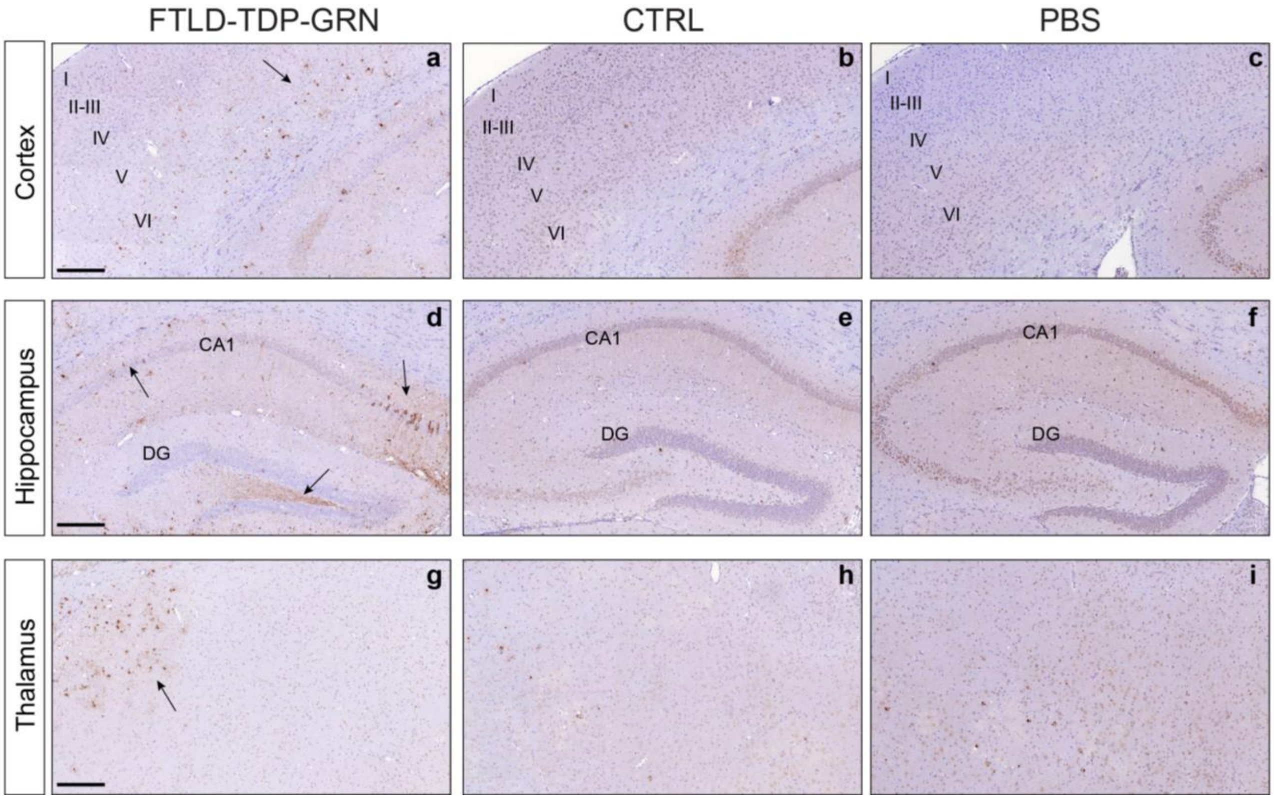

Supplementary Figure 8. TDP-43 seeding in rNLS8 due to FTLD-TDP-GRN extracts and not control extracts or PBS. Representative photomicrographs of p409-410 IHC staining in cortex (layers I-VI, **a**-**c**), hippocampus (**d**-**f**) and thalamus (**g**-**i**) of rNLS8 mice following brain injections with FTLD-TDP-GRN (n=3), age matched non-pathological (CTRL) (n=3) extracts or PBS (n=2) at 1 mpi. IHC p409-410 staining detected aggregate bearing neurons in cortex (**a**, arrow), hippocampus (CA1 and DG) (**d**, arrows) and thalamus (**g**, arrow) in rNLS8 mice injected with FTLD-TDP-GRN extracts, but not in mice injected either with CTRL extracts (**b, e** and **h**) or PBS (**c, f** and **i**). Scale bar = 200 µm (**a**-**c**, **d**-**f** and **g**-**i**)

Supplementary Figure 9

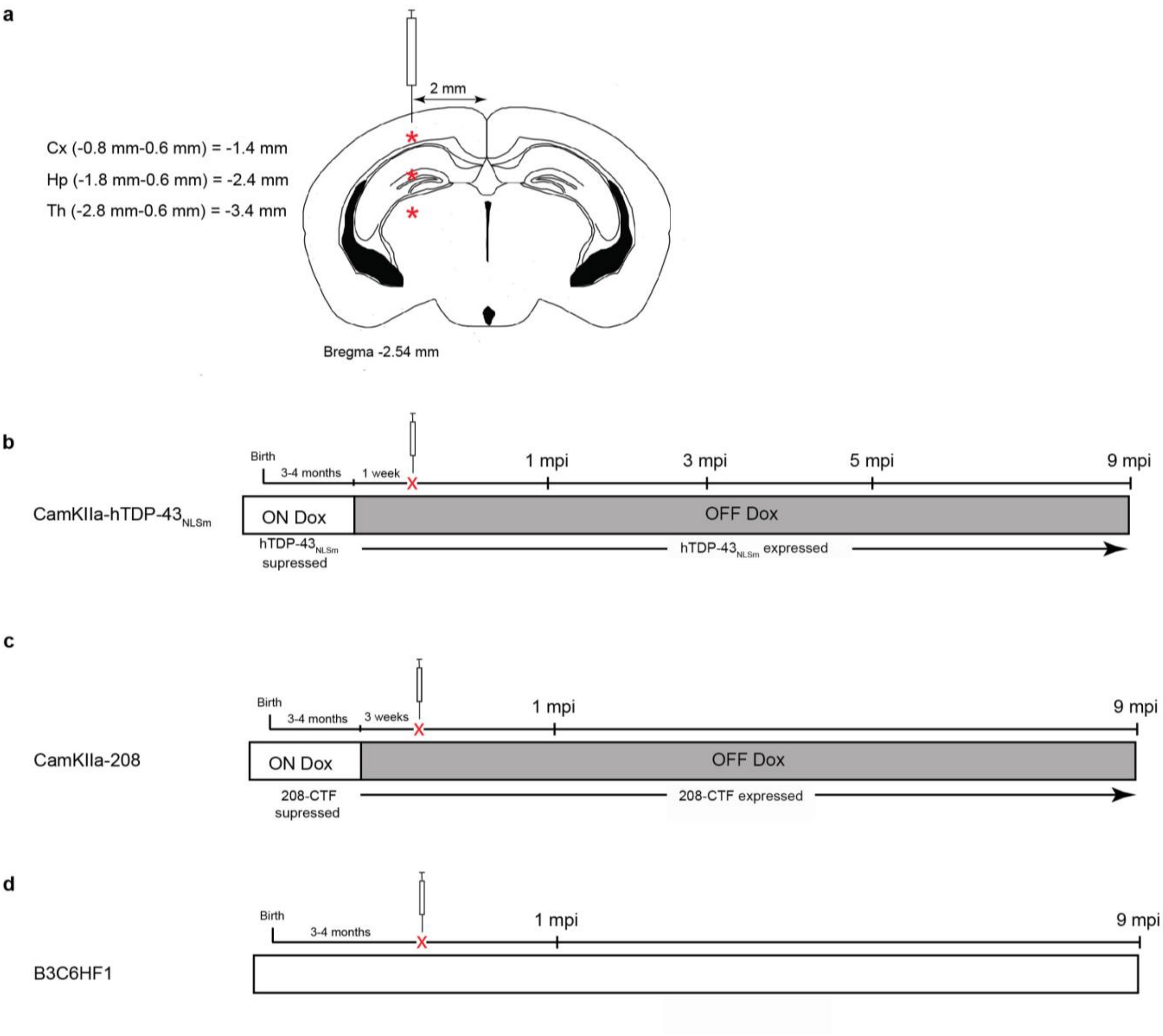

Supplementary Figure 9. Experimental design to assess time dependent spreading of TDP-43 pathology. Schematic illustrations of the injection sites (**a**) as well as experimental paradigm for CamKIIa-hTDP-43$_{NLSm}$ (**b**) CamKIIa-208 (**c**) and non-Tg mice (B3C6HF1) (**d**) used in these studies.

Supplementary Figure 10

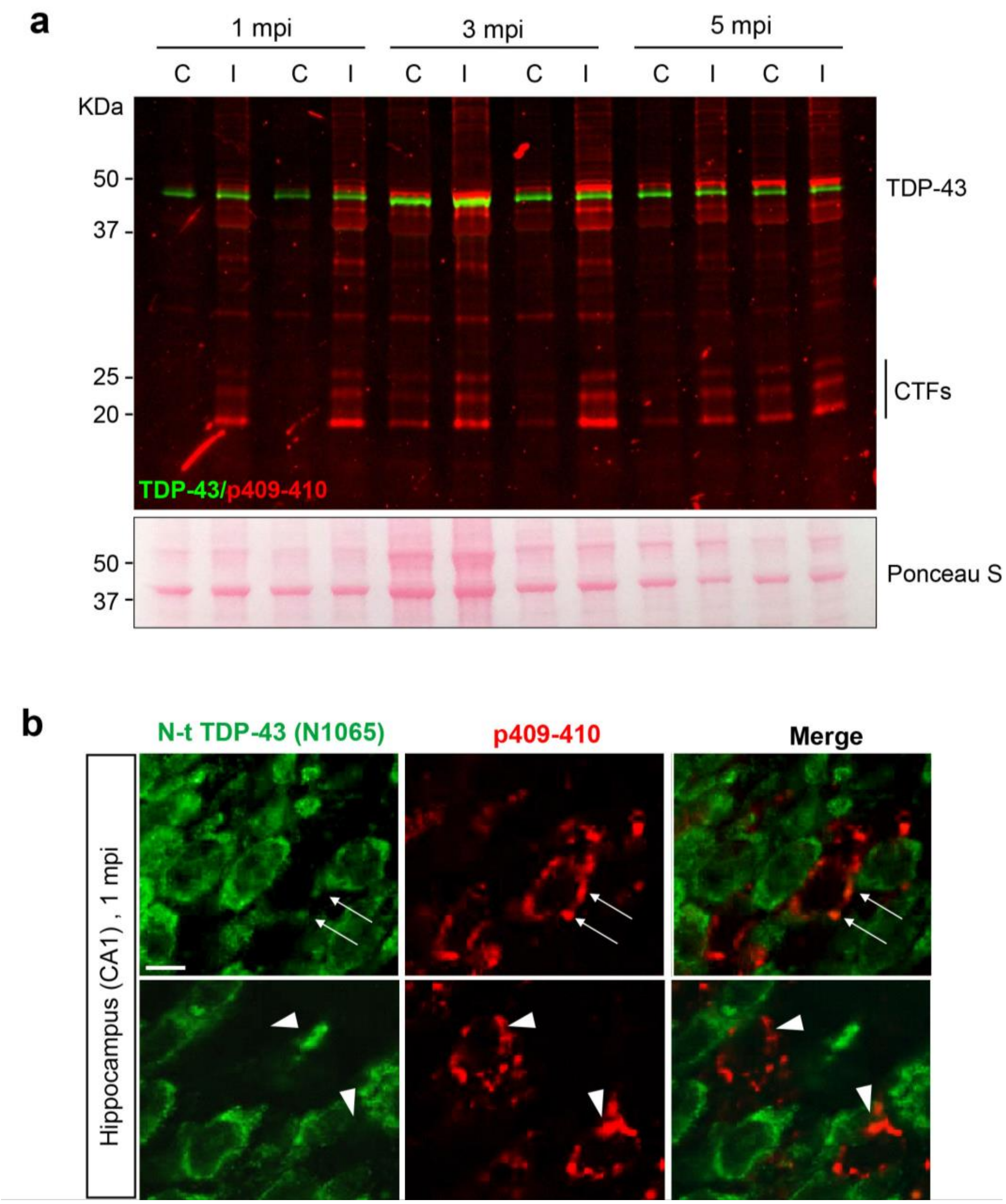

Supplementary Figure 10. Biochemical characterization of hippocampal TDP-43 pathology in injected CamKIIa-hTDP-43$_{NLSm}$ mice.

a) Immunoblot analysis of RIPA-insoluble fraction from ipsilateral (I) and contralateral (C) hippocampal homogenates from CamKIIa-hTDP-43$_{NLSm}$ mice injected with FTLD-TDP-GRN extracts at 1, 3 and 5 mpi. A C-terminus TDP-43 antibody (C1039, green) and the phospho-specific p409-410 antibody (p409-410, red) were used to detect insoluble full-length TDP-43 (~43 KDa) and CTFs (~20-25 KDa) proteins. Molecular weight markers in KDa are shown on the *left* and Ponceau S red staining was used as a loading control (bottom panel). **b**) Representative double-label IF images of p409-410 and a specific N-terminus TDP-43 (N1065) antibodies in coronal hippocampal sections from injected CamKIIa-hTDP-43$_{NLSm}$ mice at 1 mpi (n=3). Arrows point to p409-410 positive NCIs co-localizing with N-t TDP-43 immunostaining (upper panels), and arrow heads point to p409-410-positive NCI but N-t TDP-43 negative (bottom panels). Scale bar = 10 µm.

Supplementary Figure 11

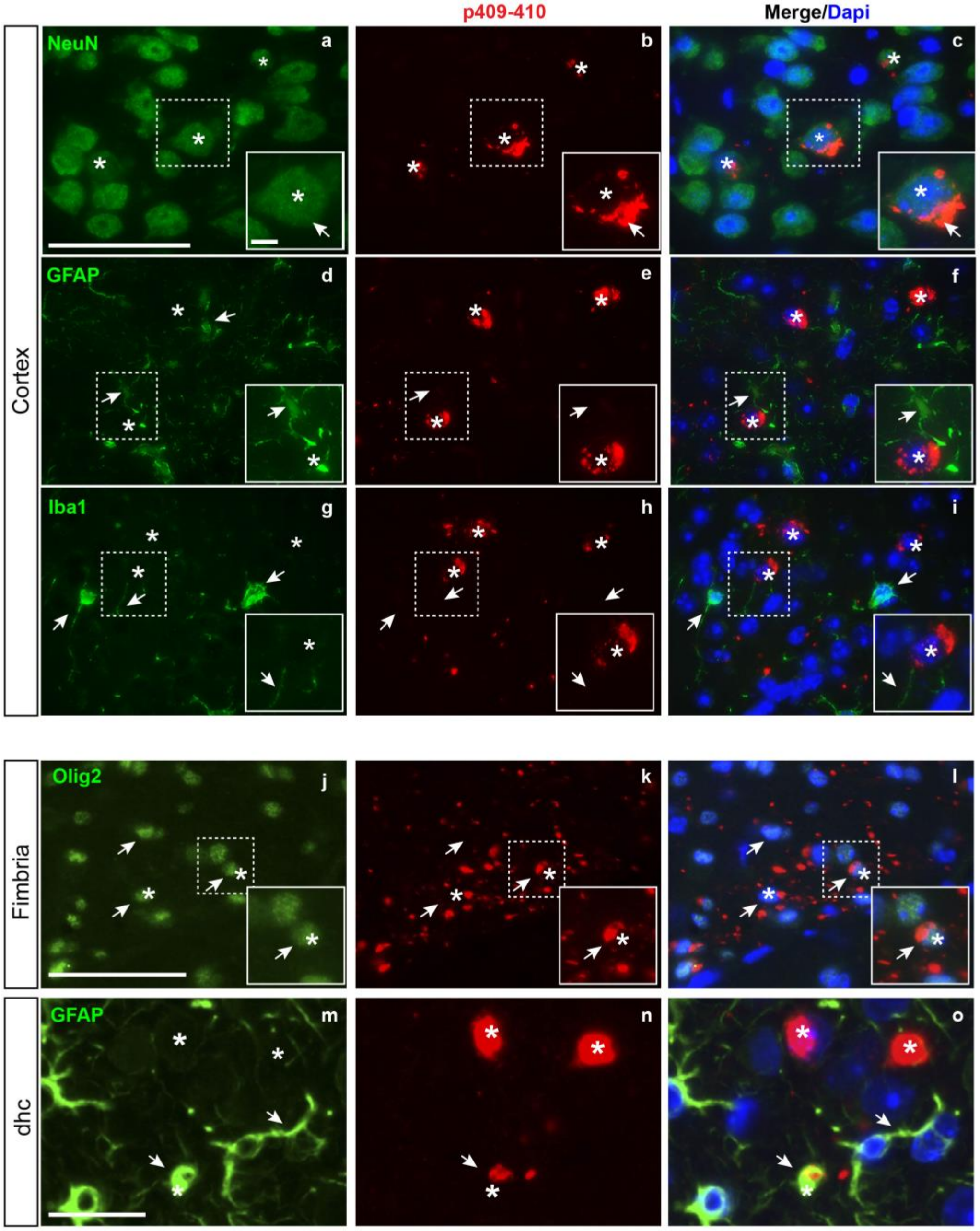

Supplementary Figure 11. p409-410 positive cytoplasmic inclusions were found in NeuN positive neurons and rarely in oligodendrocytes and astrocytes in the white matter tracts.

Representative double-label IF images of p409-410 staining (**b**-**c, e**-**f, h**-**i, k**-**l** and **n**-**o**) showing neuronal (NeuN, **a** and **c**), astrocytic (GFAP, **d** and **f**) and microglial (Iba1, **g** and **I)** markers in the cortex, oligodendroglia (Olig2, **j** and **l**) in the fimbria and GFAP (**m** and **o**) in the dorsal hippocampal commissure (dhc) of injected CamKIIa-hTDP-43$_{NLSm}$ mice at 9 mpi (n=3). Asterisks indicate cells with p409-410 positive cytoplasmic aggregates (**a**-**o** and insets), arrows indicate cells positive for NeuN (**a**-**c** and insets), GFAP (**d**-**f**, **m**-**o** and insets), Iba1 (**g**-**i** and insets) and Olig2 (**j**-**l** and insets). Insets are higher magnifications of the white-dashed boxes in **a**-**l**. Brain sections were counterstained with DAPI to label the nuclei (**c**, **f, i**, **l** and **o**). Scale bar = 50 µm (**a**-**i**, **j**-**l** and **m**-**o**) and 10 µm (insets).

Supplementary Figure 12

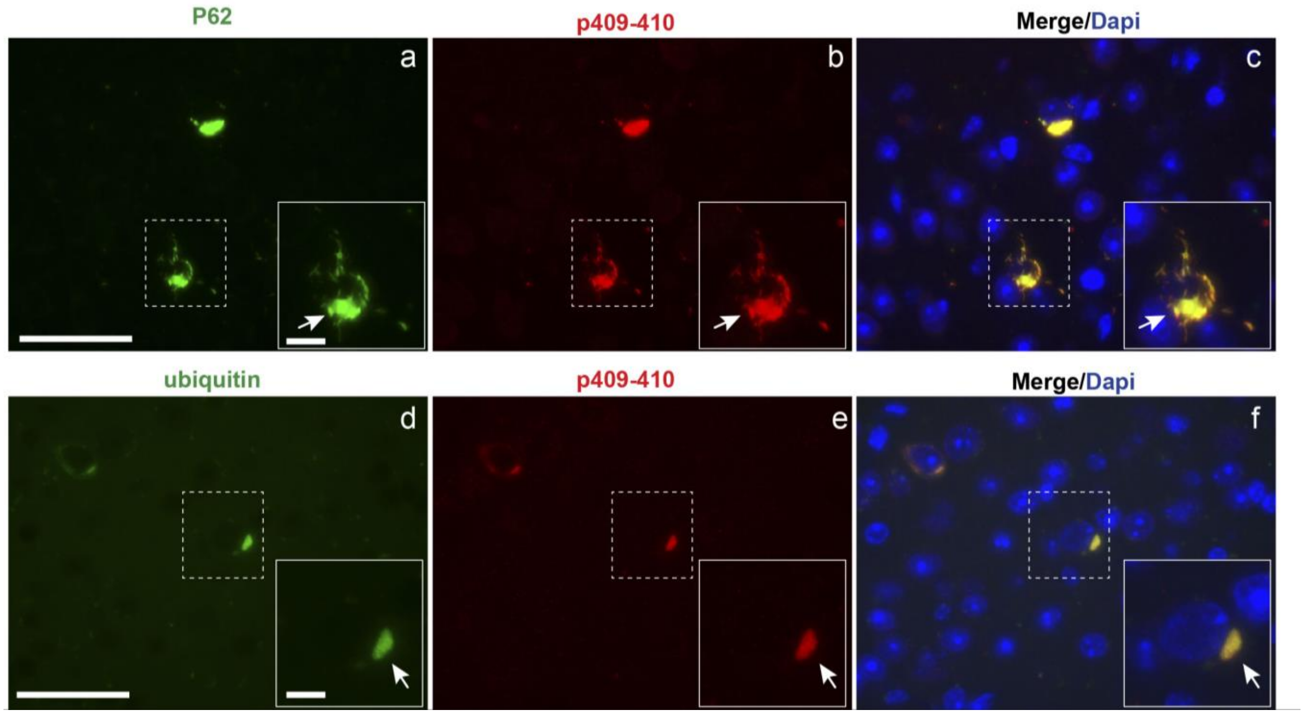

Supplementary Figure 12. p409-410 positive NCIs co-localize with p62 and ubiquitin.

Representative double-label IF images of p409-410 (**b**-**c** and **e**-**f**) and P62 (**a** and **c**) and ubiquitin (**d** and **f**) staining in cortex of injected CamKIIa-hTDP-43$_{NLSm}$ mice at 1 mpi (n=3). Insets are higher magnifications of white-dashed boxes in **a**-**c** and **d**-**f**. Arrows point to cells with p409-410 positive inclusions. Brain sections were counterstained with DAPI to label the nuclei (**c** and **f**). Scale bar = 50 µm (**a**-**c** and **d**-**f**) and 10 µm (insets).

Supplementary Figure 13

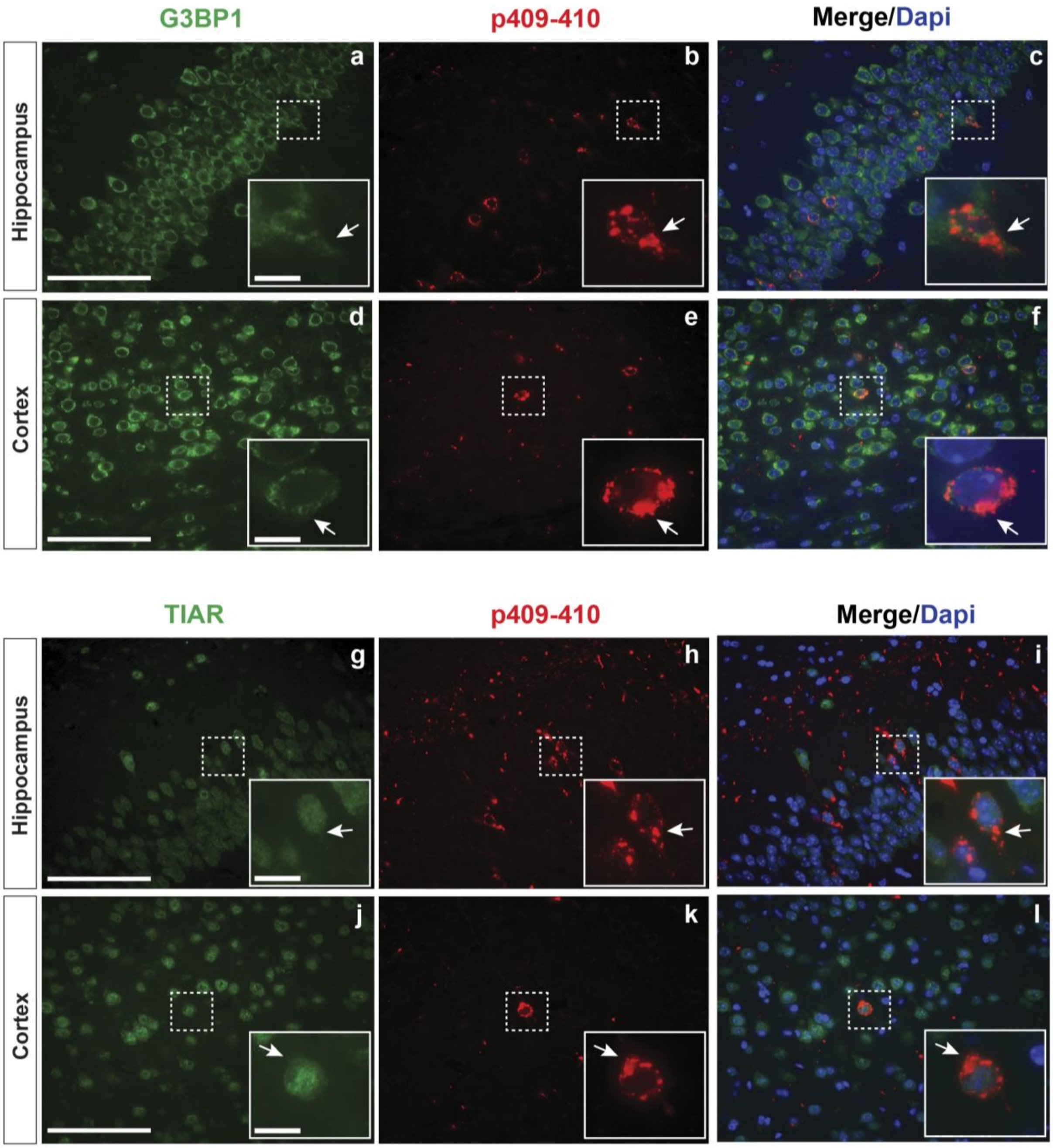

Supplementary Figure 13. p409-410 positive NCIs do not co-localize with the stress granule markers G3BP1 or TIAR Representative double-label IF images of p409-410 (**b**-**c**, **e**-**f**, **h**-**i** and **k**-**l**) and G3BP1 (**a**, **c**, **d** and **f**) and TIAR (**g**, **i**, **j** and **l)** in hippocampus and cortex of injected CamKIIa-hTDP-43$_{NLSm}$ mice at 1 mpi (n=3). Insets are higher magnifications of the white-dashed boxes in **a**-**l**. Arrows point to cells with p409-410 positive NCIs. Brain sections were counterstained with DAPI to label the nuclei (**c**, **f**, **i** and **l**). Scale bar = 100 µm (**a**-**c**, **d**-**f**, **g**-**l** and **j**-**l**) and 10 µm (insets).

Supplementary Figure 14

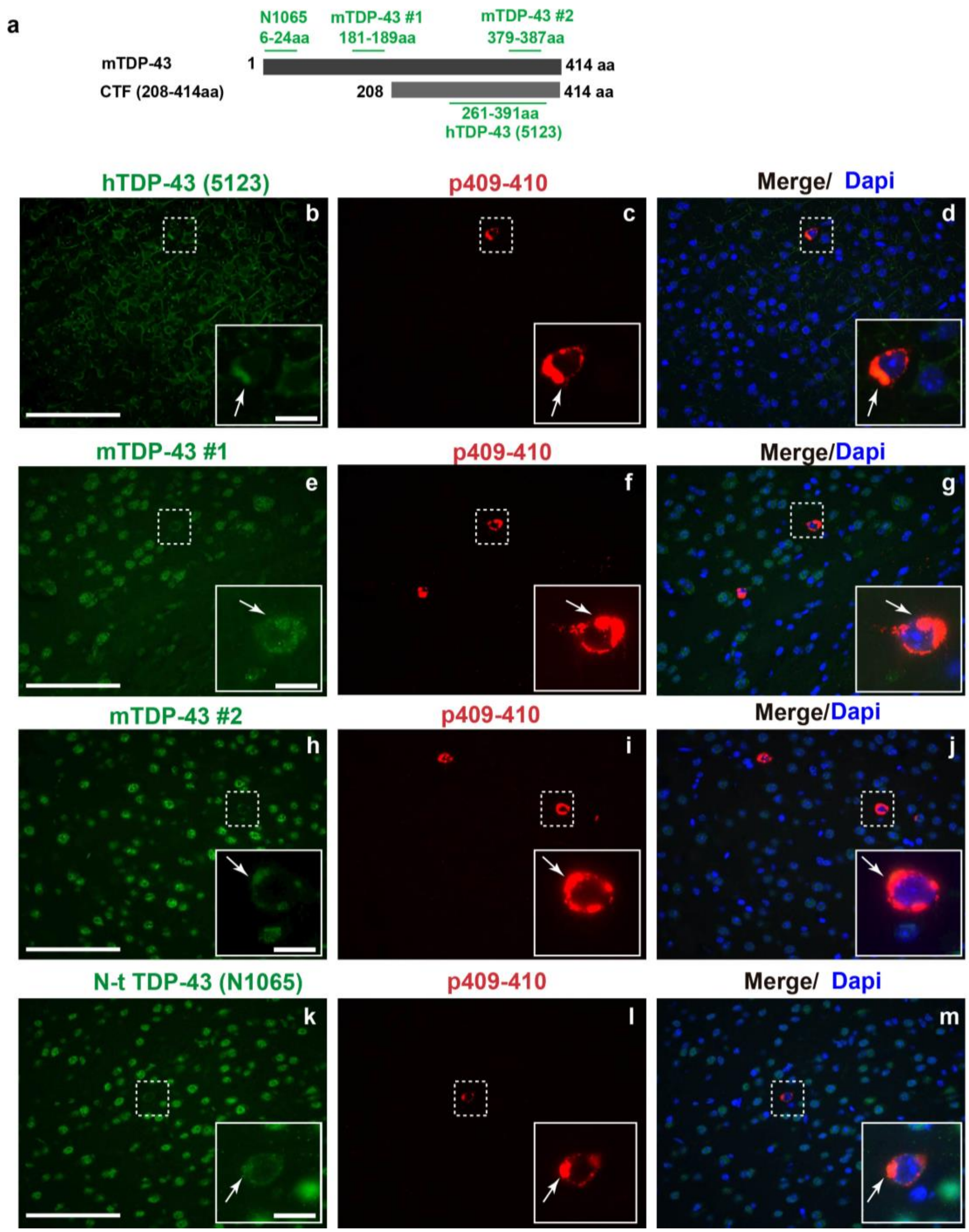

Supplementary Figure 14. 208-CTFs and endogenous TDP-43 are recruited into p409-410 positive aggregates in CamKIIa-208 injected mice.

a) Schematic representation of the epitopes recognized by antibodies specific for TDP-43 used to distinguish the 208-CTF transgene (hTDP-43 (5123)), endogenous mouse TDP-43 (mTDP-43 #1 and #2) and an N-terminus specific antibody (N1065). **b**) Representative double-label IF images of p409-410 (**c**-**d**, **f**-**g**, **i**-**j** and **l**-**m**) with 5123 (**b** and **d**), mTDP-43 (mTDP-43 #1 (**e** and **g**) and mTDP-43 #2 (**h** and **j**)) and N1065 (**k** and **m**) in cortex of injected CamKIIa-208 mice at 9 mpi (n=3). Insets are higher magnifications of the white-dashed boxes in **b-m** and arrows point to cells with p409-410-positive NCIs. Brain sections were counterstained with DAPI to label the nuclei (**d**, **g**, **j** and **m**). Scale bar = 100 µm (**b**-**d**, **e**-**g**, **h**-**j** and **k**-**m**) and 10 µm (insets).

Supplementary Figure 15

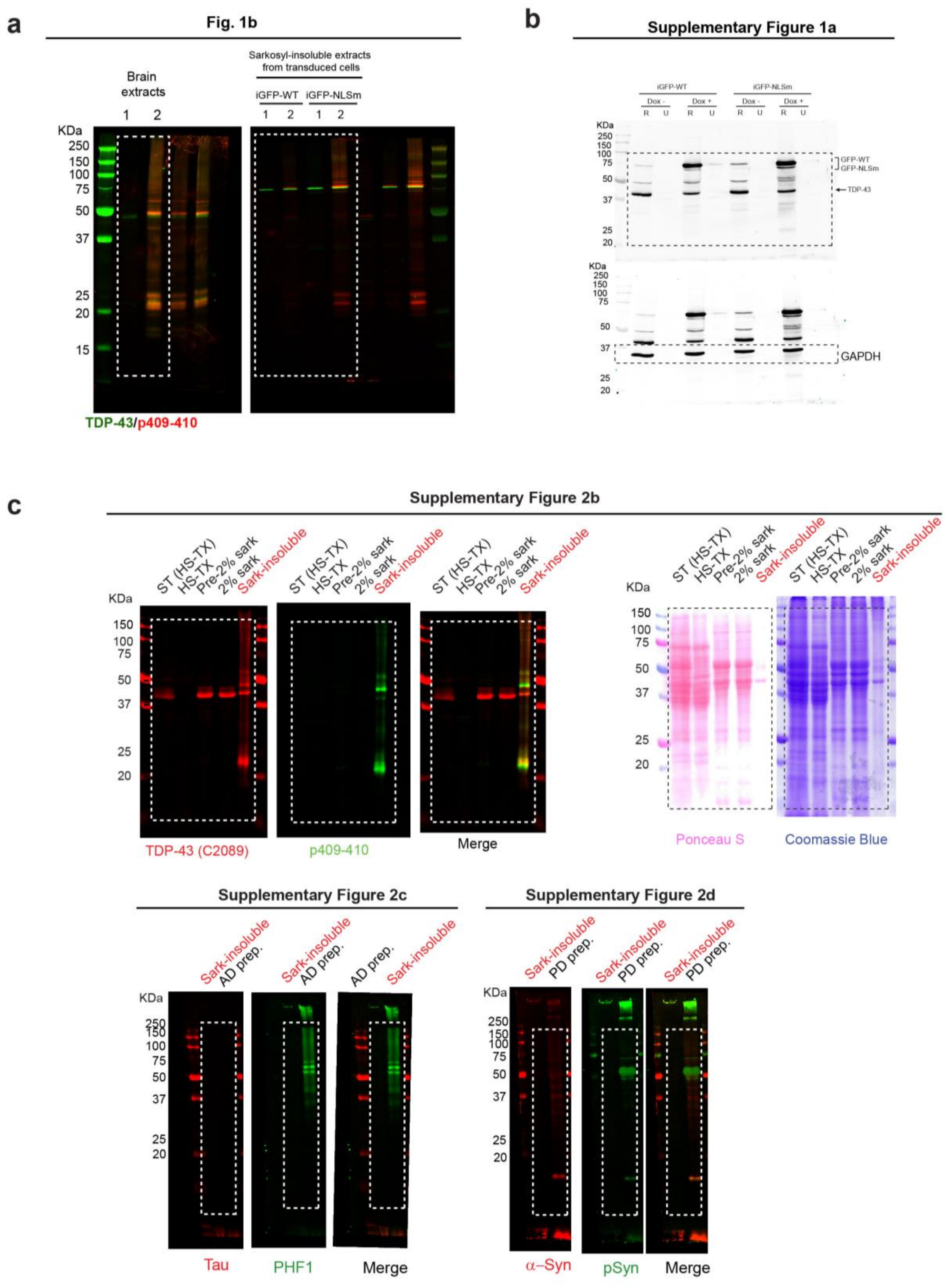

d

Supplementary Figure 3a

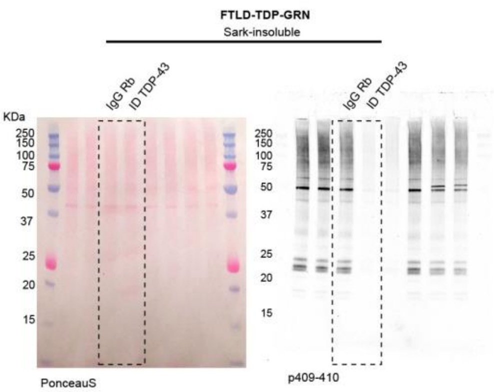

Supplementary Figure 3b

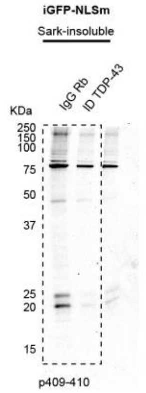

e

Supplementary Figure 4a

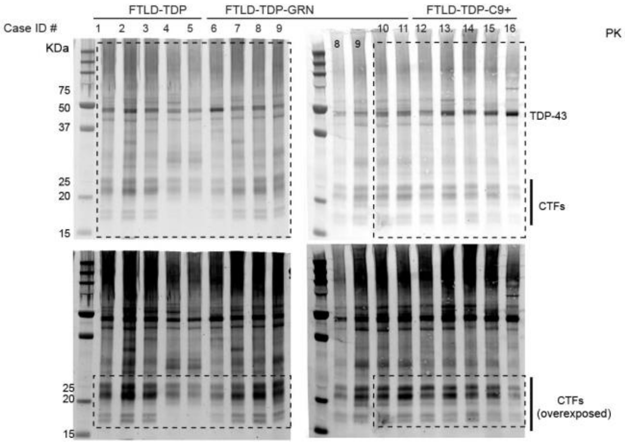

Supplementary Figure 4c

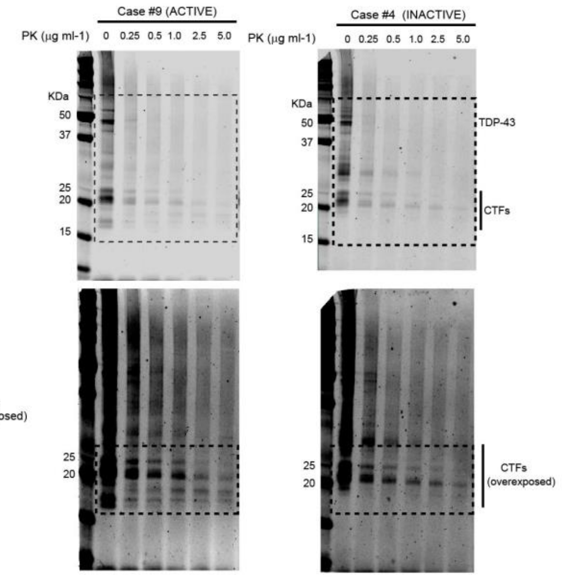

Supplementary Figure 4e

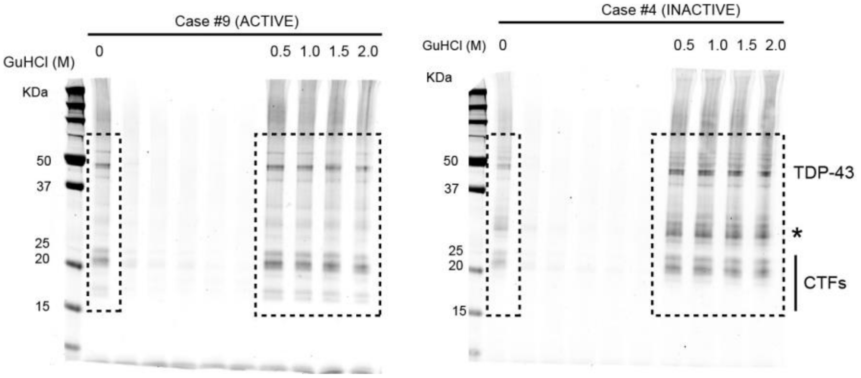

f

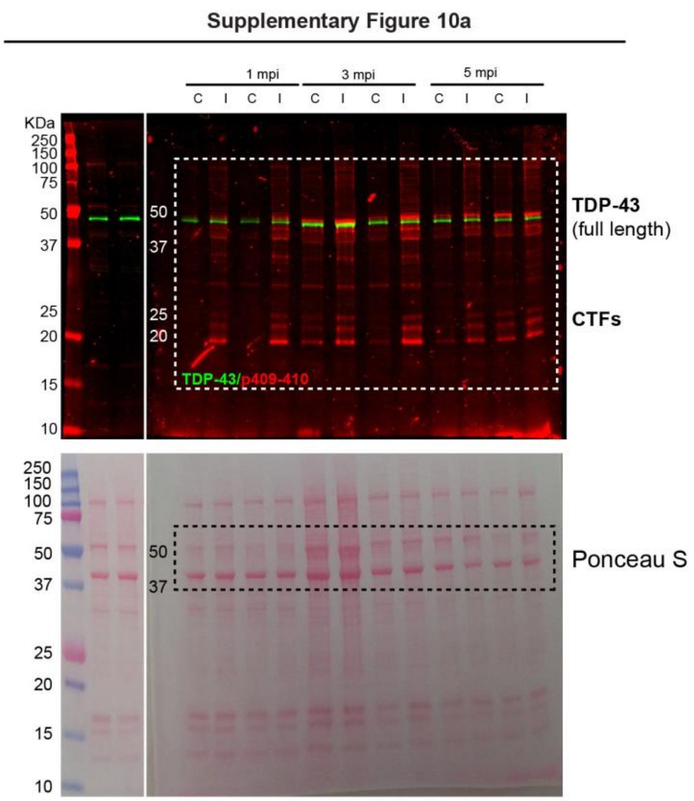

Supplementary Figure 15. Raw western blot data.

Full scan of blots and gels for Figure 1b (**a**), Supplementary Figure 1a (**b**), Supplementary Figure 2b-d (**c**), Supplementary Figure 3a-b (**d**), Supplementary Figure 4a, c and e (**e**) and Supplementary Figure 10a (**f**).

Supplementary Table 1. Genetic and demographic data of human cases used in these studies

Case No.	Neuropathological Diagnosis	Clinical Phenotype	Genetic	FTLD-TDP Subtype *	Gender	Age at onset	Age at death
1	FTLD-TDP	bvFTD	-	A	F	NA	82
2	FTLD-TDP	bvFTD	-	A	F	66	74
3	FTLD-TDP	PPA (Logopenic)	-	A	F	67	73
4	FTLD-TDP	FTLD-NOS	-	A	M	51	55
5	FTLD-TDP	PSP	-	A/B	F	67	69
6	FTLD-TDP	PPA (Logopenic)	GRN c.1414-2A>G, p.A472VfsX10	A	F	52	62
7	FTLD-TDP	bvFTD	GRN c.348A>C (splice site), p.S116S	A	M	67	78
8	FTLD-TDP	bvFTD	GRN p.R493X	A	F	54	58
9	FTLD-TDP	bvFTD	GRN c.154delA, p.T52HfsX2	A	F	62	69
10	FTLD-TDP	naPPA	GRN c.675_676delCA, p.Ser226TrpfsX28	A	M	56	66
11	FTLD-TDP	Corticobasal syndrome	GRN c.1317_1318delCA, p.Asp441HisfsX4	A	M	50	55
12	FTLD-TDP	bvFTD	C9orf72 Expansion	A	M	53	60
13	FTLD-TDP	FTLD-NOS	C9orf72 Expansion	B	M	70	75
14	FTLD-TDP	bvFTD	C9orf72 Expansion	B	M	71	77
15	FTLD-TDP	FTLD-NOS	C9orf72 Expansion	B	F	62	70
16	FTLD-TDP	bvFTD	C9orf72 Expansion	A/B	M	64	76
17	CTRL	-	-	-	F	-	60
18	CTRL	-	-	-	F	-	67

* Classification system (Mackenzie et al. 2011 and Mackenzie et al. 2017)[1,2]

Abbreviations: bvFTD - behavioral variant frontotemporal degeneration, PSP- Progressive supranuclear palsy, PPA - Primary progressive aphasia, naPPA - non-fluent/agrammatic variant of PPA, svPPA - semantic variant of PPA.

Supplementary Table 2. ELISA analysis of TDP-43 protein content and BCA measures in sarkosyl-insoluble extracts from sporadic and familial FTLD-TDP used in the *in vitro* studies

Case No.	Genetic	ng TDP-43 (C89)* ml^{-1}	ng TDP-43 (N65)** ml^{-1}	BCA (mg ml^{-1})
1	-	428.0	482	2.16
2	-	237.3	181	1.70
3	-	796.9	848	2.28
4	-	874.2	268	1.52
5	-	752.0	491	1.47
6	GRN c.1414-2A>G, p.A472VfsX10	569.8	570	2.72
7	GRN c.348A>C (splice site), p.S116S	419.0	385	1.38
8	GRN p.R493X	1045.4	763	2.40
9	GRN c.154delA, p.T52HfsX2	447.9	451	1.76
10	GRN c.675_676delCA, p.Ser226TrpfsX28	514.2	572	3.57
11	GRN c.1317_1318delCA, p.Asp441HisfsX4	806.7	629	3.76
12	C9orf72 Expansion	397.5	417	1.10
13	C9orf72 Expansion	569.2	958	3.43
14	C9orf72 Expansion	511.1	895	1.88
15	C9orf72 Expansion	381.1	698	2.86
16	C9orf72 Expansion	521.4	716	3.00

* ELISA measure of TDP-43 content using a C-terminus anti-TDP-43 antibody (C89) as a reporter.

** ELISA measure of TDP-43 content using a N-terminus anti-TDP-43 antibody (N65) as a reporter.

Supplementary Table 3. Summary of sarkosyl-insoluble human material used in the *in vivo* injection studies

	Sarkosyl-insoluble human material injected					
**Mouse Line**	**Diagnostic (Case No.)**	**[TDP-43]* ng µl^{-1}**	**ng TDP-43 per site #**	**[total protein]** µg µl^{-1}**	**µg total protein per site #**	**Time post injection**
**CamKIIa-hTDP-43$_{NLSm}$**	FTLD-TDP (#2)	~ 0.27	~ 0.67	~ 2.3	~ 5.75	1 mpi
	FTLD-TDP-GRN (#12)	~ 0.32	~ 0.79	~ 1.55	~ 3.87	1 mpi
	FTLD-TDP-GRN (#11)	~ 0.55	~ 1.25	~ 5.4	~ 13.5	1 mpi
	FTLD-TDP-C9+ (#14)	~ 0.45	~ 1.12	~ 1.4	~ 3.5	1 mpi
	FTLD-TDP-GRN (#11)	~1.1	~ 2.75	~ 7.2	~18.0	1 mpi, 3 mpi, 5 mpi, 9 mpi
	CTRL	~ 0.14	~ 0.35	~ 4.97	~12.4	1 mpi
**CamKIIa-208**	FTLD-TDP-GRN (#11)	~ 0.55	~ 1.25	~ 5.4	~13.5	1 mpi, 9 mpi
**B3C6HF1**	FTLD-TDP-GRN (#11)	~ 0.55	~ 1.25	~ 5.4	~ 13.5	1 mpi, 9 mpi
**rNLS8**	FTLD-TDP-GRN (#11)	~ 0.55	~ 1.25	~ 5.4	~ 13.5	1 mpi
	CTRL	~ 0.04	~ 0.09	~ 3.6	~ 9.0	1 mpi

* ELISA measure of TDP-43 content using a C-terminus anti-TDP-43 antibody (C89) as a reporter.

** BCA quantification.

Stereotaxic injection in Cortex, Hippocampus and Thalamus (2.5 µl extract per site).

Abbreviations: mpi - months post-injection.

Supplementary Table 4. Antibodies used in these studies

Name antibody	Epitope	Source (Cat. #)	Host	Method (dilution or concentration)			
				IHC	IF	IB	ELISA
**5104**	human TDP-43 (aa 261-391)	In-house [3]	Mo (IgG1)	0.016 µg ml^{-1}		0.10 µg ml^{-1}	
**5123**	human TDP-43 (aa 261-391)	In-house [3]	Mo (IgG1)		0.01 µg ml^{-1}		
**N1065**	TDP-43 (aa 6-24)	In-house [4]	Rb		0.20 µg ml^{-1}		1.0 µg ml^{-1}
**205**	TDP-43 (aa 262-391)	In-house [3]	Mo (IgG2a)				5.0 µg ml^{-1}
**C1039**	TDP-43 (aa 394-414)	In-house [5]	Rb		0.33 µg ml^{-1}	0.33 µg ml^{-1}	
**C2089**	TDP-43 (aa 394-414)	In-house	Rb			0.16 µg ml^{-1}	0.2 µg ml^{-1}
**mTDP-43 #1**	mouse TDP-43 (aa 181-189)	In-house [4]	Rb		0.04 µg ml^{-1}		
**mTDP-43 #2**	mouse TDP-43 (aa 379-387)	In-house	Rb		0.01 µg ml^{-1}		
**p409-410**	pTDP-43 (phosphorylated at Ser409/Ser410)	Gift from and Dr. M. Neumann and Dr. E. Kremmer (TAR5P-1D3)	Rat	1:200	1:200	1:200	
**p409-410**	pTDP-43 (phosphorylated at Ser409/Ser410)	Cosmo Bio Co., Ltd. (TIP-PTD-M01)	Rb		1:5,000		
**p403-404**	pTDP-43 (phosphorylated at Ser403/Ser404)	Cosmo Bio Co., Ltd. (TIP-PTD-P05)	Rb	1:3,000			
**Tau (17025)**	Tau	In-house [6]	Rb			1:1,000	
**PHF-1**	ptau (phosphorylated at Ser396/Ser404)	Gift from Dr. Peter Davies	Mo (IgG1)			1:1,000	
**αSyn (SNL4)**	α-synuclein (N-terminal)	In-house [7]	Rb			1:1,000	
**pSyn (81A)**	p-synuclein (phosphorylated at Ser129)	In-house [7]	Mo (IgG2a)	1:20,000		1:1,000	
**P62**	SQSTM1	Abnova (H00008878-M01)	Mo		1:500		
**Ubiq**	ubiquitin	Millipore (MAB1510)	Mo		1:3,000		
**TIAR**	TIAR	BD Bioscience (610352)	Mo (IgG1)		1;500		
**G3BP**	G3BP	Proteintech group (13057-2AP)	Rb		1:500		
**Olig2**	Olig2	Millipore (9610)	Rb		1:250		
**GFAP**	GFAP	Dako (Z0334)	Rb		1:2,000		
**Iba1**	Iba1	Wako (019-19741)	Rb		1:1,000		
**NeuN**	NeuN	Abcam (177487)	Rb		1:1,000		
**Biotinilated anti-rat IgG (H+L)**		Vector (BA-4001)	Rb	1:300			
**Biotinilated anti-rabbit IgG (H+L)**		Vector (BA-1000)	Goat	1:1,000			
**anti-rabbit IRDye 680RD**		LI-COR Bioscience (926-68071)	Goat			1:20,000	
**anti-mouse IgG IRDye 680RD**		LI-COR Bioscience (926-32210)	Goat			1:20,000	
**anti-rat IRDye 800RD**		LI-COR Bioscience (612-132003)	Goat			1:10,000	
**anti-rat IRDye 680RD**		LI-COR Bioscience (926-68076)	Goat			1:10,000	
**anti-mouse IRDye 800RD**		LI-COR Bioscience (926-32210)	Goat			1:20,000	
**anti-rabbit IgG IRDye 800CW**		LI-COR Bioscience (926-32211)	Goat			1:20,000	
**anti-rabbit Alexa Fluor 594-conjugated**		Molecular Probes	Goat		1:1,000		
**anti-rat Alexa Fluor 594-conjugated**		Molecular Probes	Goat		1:1,000		
**anti-mouse Alexa, Fluor 488-conjugated**		Molecular Probes	Goat		1:1,000		
**anti-rabbit Alexa, Fluor 488-conjugated**		Molecular Probes	Goat		1:1,000		

Abbreviations; Ref.-reference, IHC – immunohistochemistry, IF - immunofluorescence, IB - immunoblot; ELISA – Enzyme Linked Immunosorbent Sandwich Assay

Supplementary Methods

Preparation of Sarkosyl-Insoluble Fractions from FTLD-TDP and Control Brains

Sarkosyl-insoluble extracts from human post-mortem brains were obtained by sequential extraction using buffers of increasing strength (Supplementary Fig. 2a)[8]. Briefly, grey matter from frozen frontal cortex was extracted with 5.0 ml g^{-1} tissue with high-salt buffer (HS; 10 mM Tris–HCl, pH 7.4, 0.5 M NaCl, 2 mM EDTA, 10% sucrose (w:v), and 1 mM dithiothreitol (DTT)) containing 1% Triton X-100 (HS-TX), and a cocktail of protease/phosphatase inhibitors (1 mM phenylmethylsulfonyl fluoride, a mixture of protease inhibitors (1 mg ml^{-1}, pepstatin, leuptin, N-p-Tosyl-L-phenylalanine chloromethyl ketone, Na-Tosyl-L-lysine chloromethyl ketone hydrochloride, trypsin inhibitor; Sigma, St Louis, MO) and a mixture of phosphatase inhibitors (2 mM imidazole, 1 mM NaF, 1 mM sodium orthovanadate; Sigma, St Louis, MO). Myelin was removed by floatation in HS-TX buffer containing 20% sucrose post-centrifugation. The remaining pellet was treated with BitNuclease (500 U g^{-1} tissue, Biotool Co, Houston, TX) on ice for 30 min to remove DNA and RNA before extraction with HS buffer containing 2% sarkosyl (HS-2% sark). The resulting pellet was washed in PBS (3.0 ml g^{-1} tissue) and re-suspended in PBS (0.1 ml g^{-1} tissue) by sonication using a hand-held probe (QSonica, Newtown, CT). The sequentially extracted fractions were characterized by immunoblot, Ponceau S and Coomassie Blue staining. Total protein concentration in the final sarkosyl-insoluble fractions used in seeding experiments were measured by BCA assay (Thermo Scientific Inc., Rockford, IL). TDP-43 protein concentration in the sarkosyl-insoluble fraction was determined using TDP-43 sandwich ELISA (see below).

PK Digestion and GuHCl Denaturation Assay

For PK digestion, equal amounts of total proteins ~10 µg (~ 2.5 ng of TDP-43 protein) for each samples were incubated with increasing concentrations of PK (i.e., 0.25, 0.5, 1.0, 2.5 and 5.0 µg ml^{-1}) for 30 min at 37 °C. The reaction was stopped with 1mM PMSF and samples were centrifuged at 45,000xg for 1h at 4 ºC. The pellet was resuspended in 20 µl of sample buffer (1.5x) and analyzed by immunoblot.

For GuHCl denaturation, sarkosyl-insoluble human extracts (~ 2.5 ng of TDP-43 protein) were denatured with increasing concentrations of GuHCl (i.e., 0.5, 1.0, 1.5 and 2.0 M) for 30 minutes at 37 °C. Samples were centrifuged at 45,000xg for 1h at 4 ºC and the remaining pellet was resuspended in 20 µl of sample buffer (1.5x) and analyzed by immunoblot.

Sequential Biochemical Fractionation of Mouse Brain tissue

Tissue was thawed on ice and homogenized in 9x v w^{-1} of ice-cold RIPA buffer, containing 1 mM PMSF and protease and phosphatase inhibitor cocktail. Samples were briefly sonicated and centrifuged 100,000xg for 30 min at 4 °C, and the supernatant was taken as the RIPA-soluble fraction. The pellet was washed by sonication with 5x v w^{-1} RIPA buffer and centrifuged 100,000xg for 30 min at 4 °C. The remaining pellet, the RIPA-insoluble fraction, was resuspended in 2× v w^{-1} of PBS by sonication. Protein concentrations of the RIPA soluble fractions were determined using the bicinchoninic acid protein assay (Pierce).

Depletion of TDP-43 from the Sarkosyl Insoluble Fraction by Immunoprecipitation (IP)

Twenty µl of Dynabeads® Protein G beads (Thermo Scientific Inc., Rockford, IL) were conjugated with 5 µg of anti-TDP-43 antibody (C2089) or control purified rabbit IgG (2729S, Cell Signaling Technology, Inc., Danvers, MA) following the manufacturer's instructions. Thirty µg of sarkosyl-insoluble extracts from FTLD-TDP brains were diluted with PBS (0.3 µg protein $µl^{-1}$) and incubated with either control IgG or C2089 antibodies coupled

to beads in PBS and rotated overnight at 4 ^{o}C. The unbound fraction was separated from the antibody/beads complex using a magnet and the immunodepletion of TDP-43 in the supernatant was validated by immunoblot and used to assess the seeding activity into iGFP-NLSm cells.

Enzyme-linked Immunosorbent Sandwich Assay (ELISA)

The TDP-43 concentrations in the sarkosyl-insoluble PBS fractions were estimated using the 384-well format sandwich ELISA[3]. Briefly, plates were coated with mAb 205 (Supplementary Table 4) in 0.1 M sodium carbonate, pH 9.6 overnight at 4 °C and blocked with Block-Ace buffer (AbD Serotec, Raleigh, NC) for a minimum of 3 days at 4 °C. Full-length recombinant TDP-43 protein standards (0-10 ng ml^{-1}) and sarkosyl-insoluble human brain extracts were added to the wells and incubated for 16 h at 4 °C. Plates were washed and incubated for 16 hrs at 4 °C with a rabbit polyclonal antibody against C-terminus TDP-43 (C2089) or N-terminus TDP-43 (N1065) (Supplementary Table 4). Horse-radish peroxidase conjugated goat anti-rabbit IgG and TMB peroxidase substrate system (Thermo Scientific Inc., Rockford, IL) were used for reporting.

Supplementary References

1. Mackenzie, I.R., et al. A harmonized classification system for FTLD-TDP pathology. *Acta Neuropathol* **122**, 111-113 (2011).
2. Mackenzie, I.R. & Neumann, M. Reappraisal of TDP-43 pathology in FTLD-U subtypes. *Acta Neuropathol* **134**, 79-96 (2017).
3. Kwong, L.K., et al. Novel monoclonal antibodies to normal and pathologically altered human TDP-43 proteins. *Acta Neuropathol Commun* **2**, 33-44 (2014).
4. Walker, A.K., et al. Functional recovery in new mouse models of ALS/FTLD after clearance of pathological cytoplasmic TDP-43. *Acta Neuropathol* **130**, 643-660 (2015a).
5. Walker, A.K., et al. An insoluble frontotemporal lobar degeneration-associated TDP-43 C-terminal fragment causes neurodegeneration and hippocampus pathology in transgenic mice. *Hum Mol Genet* **24**, 7241-7254 (2015b).
6. Ishihara, T., et al. Age-dependent emergence and progression of a tauopathy in transgenic mice overexpressing the shortest human tau isoform. *Neuron* **24**, 751-762 (1999).
7. Giasson, B.I., et al. A panel of epitope-specific antibodies detects protein domains distributed throughout human alpha-synuclein in Lewy bodies of Parkinson's disease. *J Neurosci Res* **59**, 528-533 (2000).
8. Lee, E.B., et al. Expansion of the classification of FTLD-TDP: distinct pathology associated with rapidly progressive frontotemporal degeneration. *Acta Neuropathol* **134**, 65-78 (2017).
